# New Fossil Lacewings Give New Insight into the Diversity of Mantispidae (Insecta: Neuroptera) from the Mid-Cretaceous Amber

**DOI:** 10.3390/life16040625

**Published:** 2026-04-08

**Authors:** Xianzhe Xiang, Peichao Chen, Dong Ren, Qiang Yang, Chaofan Shi

**Affiliations:** 1 School of Earth Sciences and Engineering, Sun Yat-Sen University, Guangzhou 510275, China; xiangxzh3@mail2.sysu.edu.cn; 2 South China Biodiversity Research Center, School of Life Sciences, Guangzhou University, Guangzhou 510006, China; peichao-chen@e.gzhu.edu.cn; 3 College of Life Sciences and Academy for Multidisciplinary Studies, Capital Normal University, Beijing 100048, China; rendong@mail.cnu.edu.cn

**Keywords:** fossil, Mantispidae, Mesozoic, Myanmar

## Abstract

Four new genera with four new species and one new combination of Mantispidae are described from the Upper Cretaceous (Cenomanian) amber of northern Myanmar: *Tholomantispa quinata* gen. et sp. nov., *Tholomantispa zhangzhiqiae* comb. nov., *Heteromantispa polytricha* gen. et sp. nov., *Trimantispa poseidoni* gen. et sp. nov., and *Tribelomantispa yangjiani* gen. et sp. nov. These species exhibit unique morphological characteristics, such as scale-like setae on forewings, unique male genitalia structure, and specialized raptorial foreleg, which provide valuable information for the study of character transformation and adaptive evolution within Mantispidae. The scale-like setae on the forewings suggests potential secondary loss in extant taxa. Similarly, the documentation of processes on the forefemur across multiple genera introduces a novel morphological trait within Mantispidae, enriching our knowledge of their structural diversity.

## 1. Introduction

The family Mantispidae (Neuroptera), commonly known as mantid lacewings, is a diverse group of predatory insects characterized by their raptorial forelegs. With a fossil record extending back to the Mesozoic, mantispids provide critical insights into the evolutionary history of Neuroptera. Over their 200-million-year history, mantispids exhibit a macroevolutionary trajectory defined by key morphological innovations, pulsed diversifications, and responses to global environmental changes in analogous ways to other lacewings [1,2].

The earliest mantispids, known as *Liassochrysa* from the Early Jurassic, possessed the plesiomorphic foreleg condition: a stout femur with multiple short spines, adapted for clamping prey [3,4,5]. Phylogenetic analyses indicate that the first major diversification pulse occurred from the Jurassic to the Early Cretaceous, a radiation potentially facilitated by ecological opportunities following the end-Triassic mass extinction [1,6].

A significant morphological transformation is documented from the mid-Cretaceous. Taxa such as *Pectispina* and *Lonchomantispa* developed a highly derived foreleg with an extremely elongated, rigid major spine on the femur [7]. Finite element analyses indicate that this morphology represented a functional shift, specializing in piercing soft-bodied prey rather than clamping, which expanded the family’s morphospace and functional disparity [1].

The Cenozoic marks the establishment of the modern fauna, with the primary radiation of extant subfamilies [7]. In contrast to the extreme specializations of the Cretaceous, the forelegs of Cenozoic mantispids exhibit the modern archetype, characterized by a major spine of a length comparable to the femoral width [7,8,9,10]. While foreleg morphology generally stabilized around a median-long spine, further functional refinements occurred [1].

The subfamily Doratomantispinae, established by Lu et al. [11], is known from the Upper Cretaceous amber deposits of northern Myanmar and includes genera such as *Paradoxomantispa* Lu et al., 2020, and *Doratomantispa* Poinar in Poinar & Buckley, 2011 [11,12]. These fossils are pivotal for understanding the morphological evolution and phylogenetic relationships within Mantispidae, particularly due to their well-preserved morphological features in amber.

This study describes four new genera and species, along with one new combination, from the Cenomanian Myanmar amber, which are related to Doratomantispinae. Notably, the discovery of scale-like setae on the forewings of *Heteromantispa polytricha* gen. et sp. nov. marks the first record of such structures in a male lacewing, which was previously known only in females of other families like Berothidae and Babinskaiidae. Additionally, the presence of processes on the forefemur in multiple genera represents a novel trait within Mantispidae. *Tholomantispa quinata* gen. et sp. nov. preserves the most complete male genitalia structure of an extinct mantispid species to date, revealing the overall structure of the gonocoxites 10 and 11. The transitional features exhibited by *Trimantispa poseidoni* gen. et sp. nov. and *Tribelomantispa yangjiani* gen. et sp. nov. provide informative insights. These findings provide new morphological data to refine the phylogenetic framework of Doratomantispinae and elucidate the evolutionary trends of mantid lacewings during the Cretaceous.

## 2. Materials and Methods

This study is based on four specimens from Myanmar amber. The amber pieces were collected in the Hukawng Valley (the state of Kachin in northern Myanmar). A map of the Hukawng Valley can be found in Figure 1 in Grimaldi et al. [13]. The volcaniclastic matrix of the amber is estimated to be ~98.79 ± 0.62 Ma, i.e., near the Albian/Cenomanian (Early/Late Cretaceous) boundary [14]. The specimens were deposited in the collections of the Key Laboratory of Insect Evolution & Environmental Changes, College of Life Sciences, Capital Normal University, Beijing, China (CNUB; Dong Ren, Curator).

The specimens were firstly subjected to surface preparation: the ambers were immersed in clean water or sequentially wet-polished using water-resistant sandpaper ranging from 500 to 10,000 grit and monitored under a stereomicroscope to avoid damage to the specimens. Afterward, the specimens were polished with polishing cloth and polishing paste until the surface was smooth. The treated specimens were then completely immersed in mineral oil to eliminate surface reflections.

Observations were performed using a Nikon SMZ1270 stereomicroscope (Nikon corporation, Tokyo, Japan). An LED Diascopic Illumination Stand was used to adjust oblique coherent contrast (OCC) illumination to enhance the contrast of specimens, highlighting details such as wing venation and genitalia. A Ring Fiber Illumination Set and a Flexible Double Arm Fiber Illumination Set were used to provide near-omnidirectional lighting to further reduce reflections or to produce targeted side lighting to highlight specific structural details.

Imaging was conducted using an iMG SC600C digital camera (iMG, Suzhou, China) with CapStudio 3.5.1 software. Focal plane stacking was applied when necessary to generate images with an extended depth of field. Raw images were only minimally adjusted for brightness and contrast without any further processing. Line drawings were drawn based on observations and photographs, then digitally traced and converted into vector graphics using Adobe Illustrator CC 2022. Figures were composed and labeled using Adobe Illustrator CC and Adobe Photoshop CS6.

Terminology in general follows Lambkin, Breitkreuz et al., Ardila-Camacho et al. and Li et al. [15,16,17,18,19]. Venational abbreviations: A1–A3, first to third branches of anal vein; C, costa; Cu, cubitus; CuA, cubitus anterior; CuP, cubitus posterior; MA, media anterior; MP, media posterior; R, radius; RA, radius anterior; RP, radius posterior; RP1, proximal-most branch of RP; Sc, Subcosta.

## 3. Results

### Systematic Paleontology

Class Insecta Linnaeus, 1758

Order Neuroptera Linnaeus, 1758

Family Mantispidae Leach, 1815

Subfamily Doratomantispinae Lu et al., 2020

Type genus: *Doratomantispa* Poinar in Poinar & Buckley, 2011

Included genera: *Doratomantispa* Poinar in Poinar & Buckley, 2011; *Paradoxomantispa* Lu et al., 2020; *Tholomantispa* gen. nov.

Genus *Tholomantispa* Xiang, Chen and Yang gen. nov.

Type species: *Tholomantispa zhangzhiqiae* comb. nov.

Included species: *Tholomantispa zhangzhiqiae* (Zhou et al. in Li et al., 2022) comb. nov.; *Tholomantispa quinata* gen. et sp. nov.

Etymology: The generic name is a combination of the prefix “*thol*-“ (Greek, meaning “dome”) and “*mantispa*” (the type genus of the family Mantispidae), in reference to the dome-shaped gonapophyses 10. Gender feminine.

Diagnosis: Major process of forefemur basally branched into two processes; anteroventral row with several short processes on distal 1/3–1/2; posteroventral row with multiple successively shorter processes; foretibia with a row of reclined prostrate setae; foretarsus with conical setae; male gonocoxites 9 bifurcate; gonapophyses 10 broad, dome-shaped, not strongly sclerotized; gonocoxites 11 (gonarcus) transversely arched, with the median lobe well-developed and bearing multiple spinous setae.

*Tholomantispa quinata* Xiang, Chen and Yang gen. et sp. nov.

**Figure 1:**
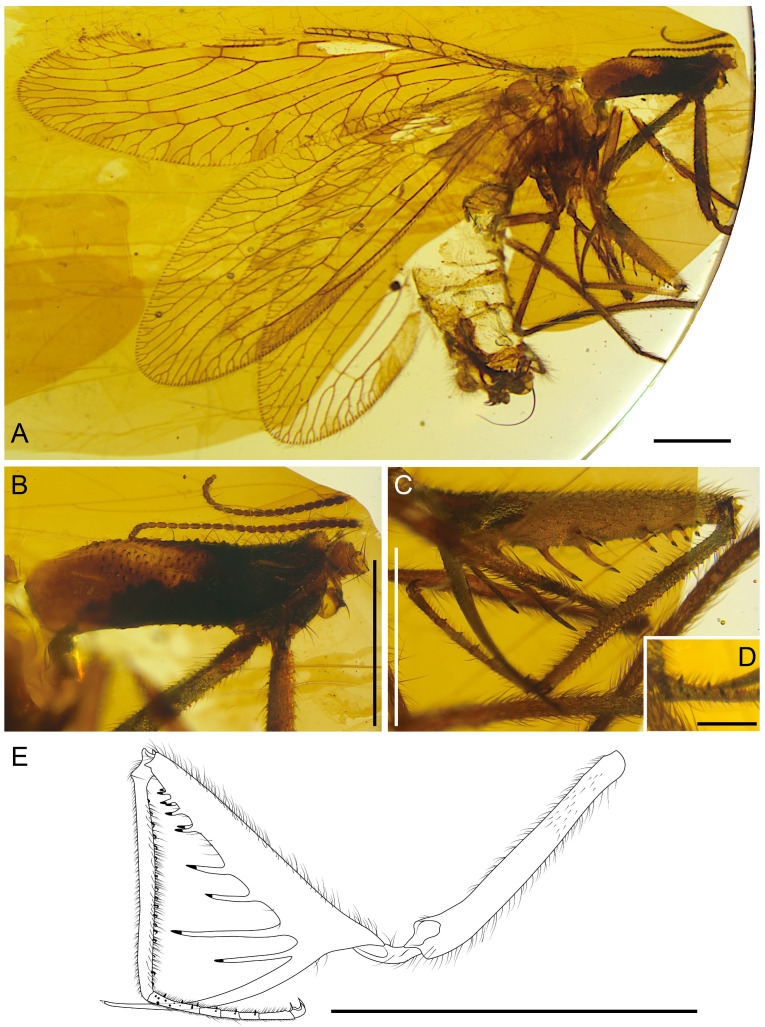
Holotype of *Tholomantispa quinata* gen. et sp. nov., CNU-NEU-MA2018085. (**A**) Habitus, lateral view; (**B**) prothorax, lateral view; (**C**) foreleg, anterior view; (**D**) protarsus, ventral view; (**E**) left foreleg, posterior view. Scale bars = 1 mm (**A**–**C**), 0.2 mm (**D**), 2 mm (**E**).

**Figure 2:**
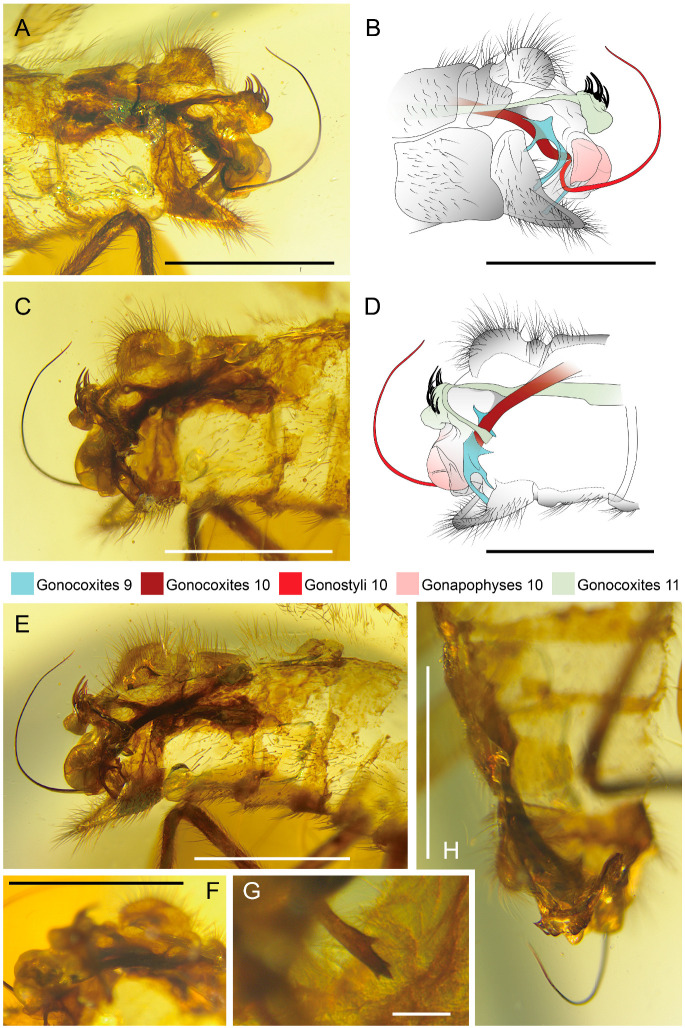
Holotype of *Tholomantispa quinata* gen. et sp. nov., CNU-NEU-MA2018085. (**A**,**B**) Male genitalia, left lateroventral view; (**C**,**D**) male genitalia, right laterodorsal view; (**E**) male genitalia, right lateral view; (**F**) basal gonocoxites 10 and gonocoxites 11, right laterodorsal view; (**G**) distal processes of left gonocoxites 9, lateral view; (**H**) gonocoxites 11, dorsal view. Scale bars = 1 mm (**A**–**F**,**H**), 0.1 mm (**G**).

**Figure 3:**
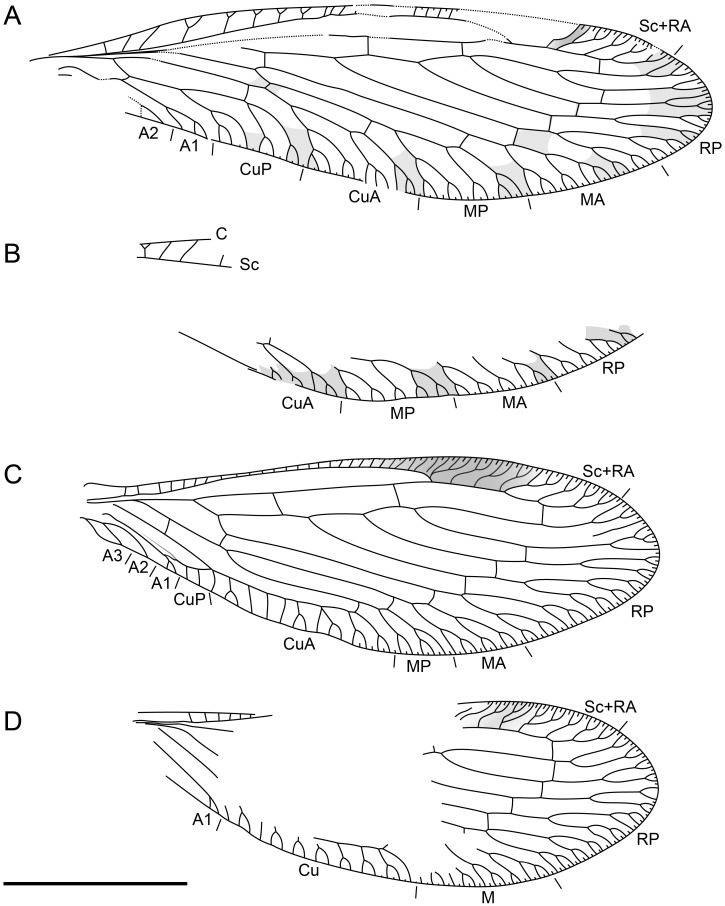
Holotype of *Tholomantispa quinata* gen. et sp. nov., CNU-NEU-MA2018085. (**A**) left forewing; (**B**) right forewing; (**C**) left hind wing; (**D**) right hind wing. Scale bar = 2 mm.

Figure 1, Figure 2 and Figure 3.

Etymology: The specific epithet is named after Latin “*quinata*” (meaning “divided into five” or “in sets of five”), in reference to the presence of five spine-like setae on each side of the gonocoxites 11 (gonarcus).

Diagnosis: Pronotum tubularly elongated, with a length/width ratio of 3.32, 1.5 times as long as the pterothorax. Forefemur major process basally branched into two processes, with the primary process 2.7 times as long as the secondary process; anteroventral row with three successively shorter processes; posteroventral row with seven successively shorter processes, the longest process 2.3 times as long as the following one; foretibia with a row of reclined prostrate setae (ca. 18); tarsomeres 1–4 of foretarsus ventrally bearing conical setae. Forewing with block-like markings, three ra-rp crossveins present, CuA with three main branches, CuP with three subpectinate branches; A1 multifork with three branches; hind wing with one gradate series, CuA pectinate with ca. nine main branches; A1 bifurcate, A2 and A3 simple. Male sternum 9 twice as long as ectoproct, distally surpassing tergum 9; gonocoxites 9 bifurcate, with basal branch distally slender and pointed, distal branch with four distal processes; gonocoxites 10 rod-like; gonostyli 10 long and slender, curved dorsad; gonapophyses 10 paired, broad, dome-shaped; gonocoxites 11 (gonarcus) transversely arched, with median lobe well-developed and bearing 10 spinous setae, median lobe with broad posteroventral projection.

Holotype: CNU-NEU-MA2018085, male, an almost complete and well-preserved specimen with genitalia intact, head and distal parts of mid- and hind legs not preserved.

Description: Antennal scape and pedicel not preserved; left antenna with 18 preserved flagellomeres, right antenna with 25 preserved flagellomeres; flagellomeres successively shorter from base to apex.

Pronotum tubularly elongated, robust, 1.5 times as long as pterothorax, anteriorly slightly expanded, with the paratergal lobes ventrally fused, covered with thick pedicellate setae, lacking maculae; mesoscutum with round anterolateral margins. Forelegs raptorial, positioned at the anterior part of pronotum; episterum and posterior cervical sclerite visible; forecoxa elongated, subequal in length to forefemur, covered with sparse fine setae and pedicellate setae; foretrochanter conical, covered with sparse thick setae; forefemur thickened, with length/width ratio 4.66, basally broad, dorsally covered with long fine setae, forefemoral closing surface with sparse setae, shorter than combined length of foretibia and foretarsus, lacking posteroventral carina; forefemoral processes saber-like, with conical Stitz organ at apex; anteroventral row with major process at proximal 3/16 length of forefemur; three anteroventral and seven posteroventral processes; foretibia straight, strongly curved basally, not produced distally, lacking tibial spur, with ventral keel and long fine setae, dorsally covered with dense short setae; foretarsus 5-segmented, with basitarsus and tarsomere 5 longer, about twice as long as tarsomeres 2–4 individually, basitarsus slightly longer than tarsomere 5, tarsomeres 2–4 subequal in length, basitarsus shorter than the combined length of tarsomeres 2–4 (ca. 0.77 times latter); tarsomeres 1–4 ventrally bearing 11, two, two, and two conical setae, respectively; pretarsus of foreleg with a pair of simple claws, arolium triangular, apically tapered; combined length of meta-femur and meta-tibia ca. 0.55 times forewing length.

Wings broad, elongate-oval (forewing length/width ratio 3.80, hind wing length/width ratio 2.92), with trichosors along entire margin except wing base, lacking nygmata and vesicae. Forewing with block-like markings near the apex and posterior margin; humeral veinlet simple; costal space basally broad, apically tapered, with 16 subcostal veinlets, most forked; Sc fused with RA distally; pterostigma present between C and Sc+RA; outer gradate series present, with gradate crossveins located proximal to primary branching points of each RP branch; 1r-m crossvein between RP stem and MA; MA subpectinately branched with three branches, MP subpectinately branched with three branches, distally bifurcate or multifurcate; ma-mp crossvein present; two m-cu crossveins detected; Cu deeply forked; one cua-cup crossvein between the distal stem of CuA and the distal branch of CuP; CuA subpectinately branched with three main branches, branches distally multifurcate; CuP with three subpectinate branches, branches distally multifurcate or bifurcate; cup-a1 crossvein present; two anal veins detected; A1 multifurcate with three branches; A2 possibly bifurcate.

Hind wing lacking markings; pterostigma distinct, subtrapezoidal, contacting RA, with bifurcate veinlets in pterostigmal area; subcostal veinlets dense and simple, ca. 22 proximal to pterostigma; Sc fused with RA distally; sc-r crossvein absent; RP originating at ca. 3/16 of wing length, with five main RP branches, branches distally multifurcate; three ra-rp crossveins; one gradate series (outer gradate series), with most gradate crossveins located proximal to primary branching points of each RP branch; 1r-m crossvein between RP1 and M stem; two m-cu crossveins; CuA pectinate with ca. nine main branches, bifurcate or simple; CuP stem indistinct, distally bifurcate; three anal veins, A1 bifurcate, A2 and A3 simple; cup-a1 crossvein between CuP stem and A1 stem; a1–a2 and a2-a3 crossveins absent.

Abdomen covered with long thick setae; tergum 9 narrow, not fused with ectoproct; ectoproct paired, with callus cerci; sternum 9 twice as long as ectoproct, distally surpassing ectoproct; gonocoxites 9 bifurcate, with basal branch distally slender and pointed, distal branch with four distal processes; gonocoxites 10 rod-like, distally connected to base of gonostyli 10 (parameres), contacting gonapophyses 10; gonostyli 10 long and slender, curved dorsad; gonocoxites 11 (gonarcus) transversely arched, with median lobe well-developed, posteroventrally with broad projection; median lobe with 10 spinous setae arranged from middle to laterals, successively shorter laterally, with five on each lateral.

*Tholomantispa zhangzhiqiae* comb. nov.

2022 *Doratomantispa zhangzhiqiae* Li et al., p: 27

Remarks: *Tholomantispa* gen. nov. is morphologically similar to *Doratomantispa* Poinar in Poinar & Buckley, 2011, but differs in genital morphology. In *Tholomantispa* gen. nov., gonocoxites 9 are bifurcate; gonapophyses 10 are broad, dome-shaped, and weakly sclerotized. In contrast, in *Doratomantispa* (except for *Doratomantispa zhangzhiqiae* Zhou et al. in Li et al., 2022), gonocoxites 9 are simple, and gonapophyses 10 are typically broad, blade-like sclerites with weakly sclerotized anterior portions, tapering posteriorly into strongly sclerotized points [19].

Based on these distinct differences in genital morphology, D. *zhangzhiqiae* is herein transferred from *Doratomantispa* to *Tholomantispa* gen. nov., and the new combination *Tholomantispa zhangzhiqiae* comb. nov. is proposed.

Subfamily incertae sedis

Genus *Heteromantispa* Xiang, Chen and Yang gen. nov.

Type species: *Heteromantispa polytricha* Xiang, Chen and Yang gen. et sp. nov.

Etymology: The generic name is a combination of the prefix “*heter*-“ (Greek, meaning dissimilar), and “*mantispa*” (the type genus of the family Mantispidae), in reference to the scale-like setae on the forewing, which are unprecedented in the family Mantispidae. Gender feminine.

Diagnosis: Pronotum tubularly elongated, robust (length/width ratio 3.49), 1.24 times as long as pterothorax, with paratergal lobes ventrally fused, lacking maculae. Forelegs raptorial, positioned at anterior part of pronotum; forecoxa longer than forefemur; forefemur slender (length/width ratio 7.28), nearly equal to the combined length of foretibia and foretarsus, lacking posteroventral carina; forefemoral processes few, saber-like, with conical Stitz organ at apex; major process located at proximal 7/16 length of forefemur, with branches at basal 1/5 and ca. 2/5 length of the major process; posteroventral row with only one process, located at proximal 11/16 length of forefemur; tibia straight, with ventral keel, ventrally with a row of reclined prostrate setae; foretarsus 5-segmented, tarsomeres 1–3 ventrally bearing conical setae, basitarsus and tarsomere 5 longer, tarsomeres 2–4 subequal in length, basitarsus nearly equal to combined length of tarsomeres 2–4; pretarsus of foreleg with a pair of simple claws, empodium triangular, apically tapered. Mid- and hind legs long, combined length of meso-femur and meso-tibia greater than 3/5 of forewing length; basitarsus extremely long, longer than half tibia, more than 2.4 times longer than combined length of tarsomeres 2–5. Wings narrow, length/width ratio greater than 3.6, with block-like markings, with a single trichosor between the apex of two veinlets on the apical wing margin, and multiple trichosors on the basal wing margin; forewing veins covered with fine setae, basal veins also with scale-like setae on longitudinal veins Sc, RA, RP, M, Cu and subcostal veinlets, with block-like markings, humeral veinlet bifurcate, subcostal veinlets simple and sparse; with one basal sc-r crossvein, Sc not fused with RA; pterostigma distinct, not contacting RA; three ra-rp crossveins; two gradate series, inner gradate incomplete; M branches with small forks near wing margin; two m-cu crossveins; two cua-cup crossveins; CuA subpectinately branched, branches distally bifurcate or simple; CuP pectinate, distal simple; three anal veins, A1 deeply bifurcate, A2 and A3 simple; hind wing without markings, pterostigma not contacting RA; veinlets in pterostigmal area indistinct; two ra-rp crossveins; one gradate series; M branches with small forks near wing margin; two ma-mp crossveins; three m-cu crossveins; CuA with seven pectinate branches, mostly simple; CuP stem indistinct; three anal veins, all simple. Male abdomen apex covered with dense long setae, ectoproct paired, posteriorly with a long flat lobe-like projection, sternum 9 long, distally reaching half of the ectoproct lobe-like projection.

Remarks: *Heteromantispa* gen. nov. has significantly reduced forefemoral processes, with only the major process and one process in the posteroventral row; major process branched near the base, with three branches; these features are consistent with *Acanthomantispa* Lu et al., 2020, from the same locality and horizon and unique to these two genera. *Heteromantispa* gen. nov. differs from *Acanthomantispa* by the presence of scale-like setae on forewing, long setae at abdomen apex, slender sternum 9, and ectoproct lacking dorsal projection, whereas *Acanthomantispa* lacks scale-like setae on the forewing and long setae at the abdomen apex, sternum 9 subtriangular, ectoproct with short dorsal projection.

*Heteromantispa polytricha* Xiang, Chen and Yang gen. et sp. nov.

**Figure 4:**
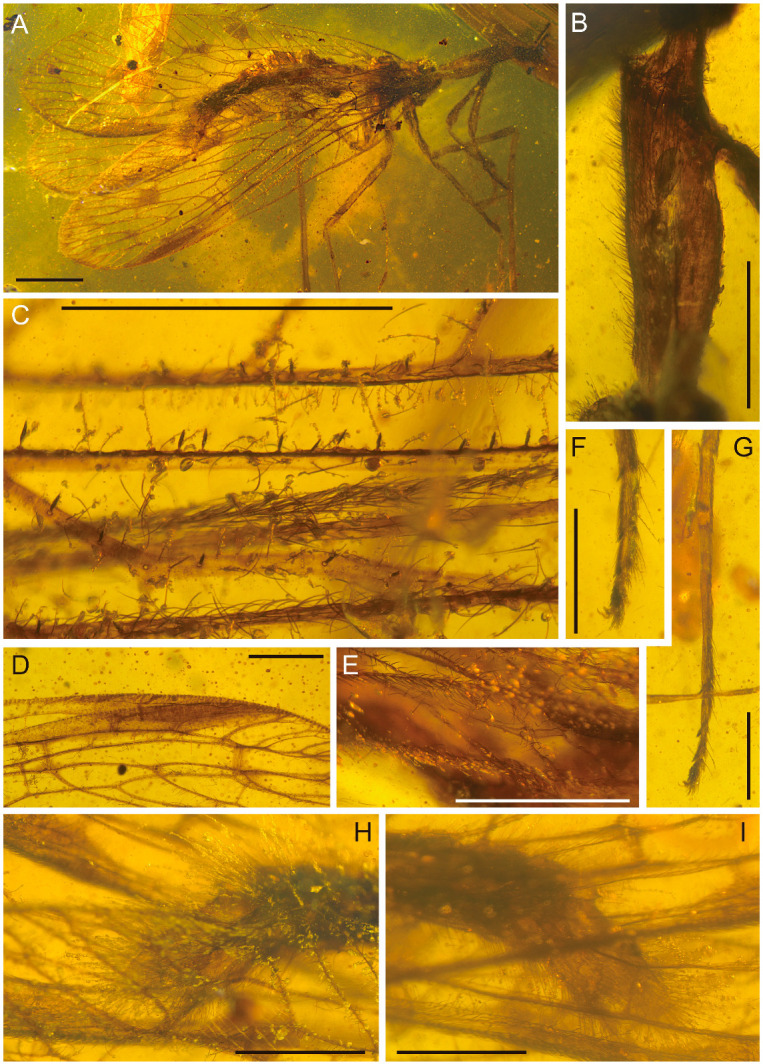
Holotype of *Heteromantispa polytricha* gen. et sp. nov., CNU-NEU-MA2018086. (**A**) Habitus, lateral view; (**B**) prothorax, lateral view; (**C**) right forewing scale-like setae; (**D**) right wing pterostigma; (**E**) right hind wing CuP; (**F**) eutarsus and pretarsus of right mid leg; (**G**) left meso-tarsus; (**H**) male genitalia, right lateral view; (**I**) male genitalia, left lateral view. Scale bars = 2 mm (**A**), 1 mm (**B**–**I**).

**Figure 5:**
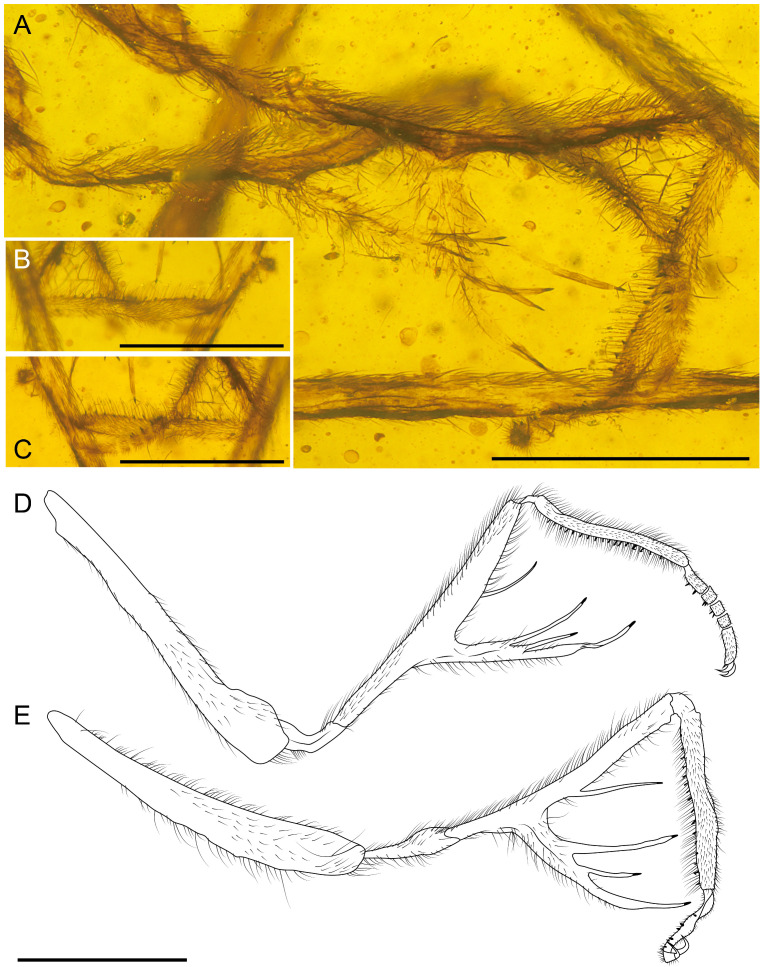
Holotype of *Heteromantispa polytricha* gen. et sp. nov., CNU-NEU-MA2018086. (**A**) Foreleg, lateral view; (**B**) right protibia, anterior view; (**C**) tarsus and pretarsus of left foreleg, posterior view; (**D**) left foreleg, anterior view; (**E**) right foreleg, posterior view. Scale bars = 1 mm.

**Figure 6:**
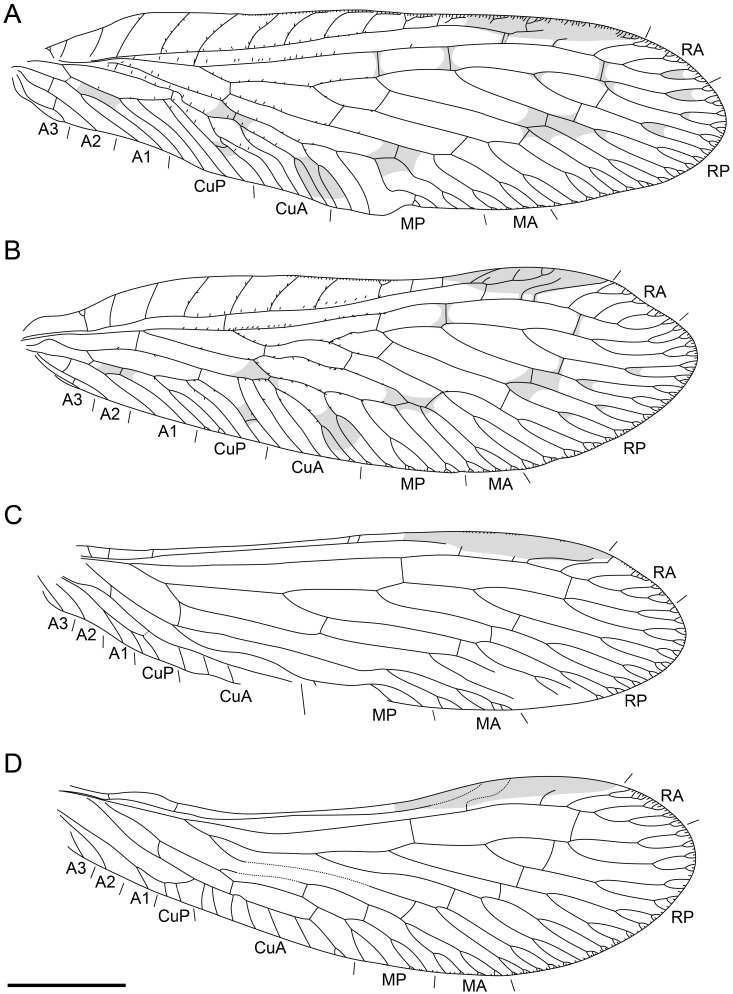
Holotype of *Heteromantispa polytricha* gen. et sp. nov., CNU-NEU-MA2018086. (**A**,**B**) Forewing; (**C**,**D**) hind wing. Scale bar = 2 mm.

Figure 4, Figure 5 and Figure 6.

Etymology: The specific epithet is named after the Latin “*polytricha*” (meaning multi-haired), in reference to the dense long setae covering the genitalia.

Diagnosis: As for the genus.

Holotype: CNU-NEU-MA2018086, male, completely preserved specimen.

Description: Head roughly triangular; compound eyes hemispherical; scape distally swollen, length/width ratio 2.45, pedicel distally not obviously swollen, length/width ratio 1.95, flagellum covered with sparse short setae, right antenna with 10 preserved flagellomeres, left with six, basal flagellomeres similar in size to pedicel.

Pronotum tubularly elongated, robust, paratergal lobes ventrally fused, covered with dense fine setae, lacking maculae; mesoscutum with round anterolateral margins. Forelegs raptorial, positioned at the anterior part of the pronotum; covered with dense fine setae; foretrochanter conical, covered with fine setae; forefemur thickened, basally elongated, dorsally covered with dense long fine setae, forefemoral closing surface with relatively sparse long setae, main stem of the major process covered with long setae; forefemur nearly equal to combined length of foretibia and foretarsus, lacking posteroventral carina; forefemoral processes significantly reduced, saber-like, with conical Stitz organ at apex; anteroventral row with major process at basal 7/16 length of forefemur, no other processes in anteroventral row; foretibia straight, basally strongly curved, distally not produced, lacking tibial spur, covered with short setae, dorsal and ventral with dense long fine setae; with ventral keel, ventrally with row of reclined prostrate setae, 17 (left foreleg) or 16 (right foreleg); foretarsus 5-segmented, basitarsus and tarsomere 5 longer, ca. 3 times tarsomeres 2–4 individually, basitarsus subequal to tarsomere 5, tarsomeres 2–4 subequal, basitarsus nearly equal to combined length of tarsomeres 2–4, tarsomeres 1–3 ventrally with three, two, and two conical setae, respectively; pretarsus of forelegs with a pair of simple claws, arolium triangular, apically tapered.

Mid- and hind legs long, combined length of femur and tibia ca. 0.60 (mid leg) or 0.74 (hind leg) of forewing length; basitarsus extremely long, 0.57 (mid leg) or 0.52 (hind leg) of tibia length, 2.4–2.7 times longer than combined length of tarsomeres 2–5; tarsomeres 2–5 successively shorter; tarsomeres 1–3 each with four setae, tarsomere 4 with eight setae visible; pretarsus with a pair of simple claws, arolium rounded.

Wings elongate-oval (forewing length/width ratio 3.65, hind wing length/width ratio 3.63), with a single trichosor between the apex of two veinlets on the apical wing margin, and multiple trichosors on the basal wing margin; forewing veins covered with fine setae. Scale-like setae straight, ca. 50–70 μm long, black and opaque, not flattened. Most scale-like setae thin at base and apex, lanceolate, some with slightly thicker base, nearly nail-like. Whether scale-like setae have longitudinal ridges is unclear. With block-like markings, costal space basally broad, apically tapered, subcostal veinlets simple, 12 (right forewing) or 10 (left forewing); 1sc-r crossvein proximal to RP origin; pterostigma distinct, nearly fusiform; Sc terminating within pterostigmal area; two veins between RA and pterostigma, basal one connected to Sc distal branch, apical one possibly RA branch; left forewing Sc distinctly terminating at C, near pterostigmal area, Sc and RA connected by one crossvein; RP originating at ca. 1/4 wing length, with six main branches, branches deeply bifurcate twice then with small forks near margin; three ra-rp crossveins; inner gradate series incomplete, outer gradate crossveins partly located distal to primary branching points of RP branches; MA terminal forking similar to RP branches; MP subpectinately branched, with 3/4 branches, branches distally bifurcate or simple, with additional small forks near the margin; ma-mp crossvein present; two m-cu crossveins, one m-cu between M stem and CuA; Cu deeply forked, two cua-cup crossveins, one cua-cup between CuA distal stem and CuP distal branch, two cua-cup between CuA basal branch and CuP distal branch; CuA subpectinately branched with four main branches, branches distally bifurcate or simple; CuP with 3/4 pectinate branches, distal simple; three anal veins, A1 deeply bifurcate, A2 and A3 simple; cup-a1, a1-a2, and a2-a3 crossveins present.

Hind wing without markings; pterostigma distinct, densely setae, nearly fusiform, not contacting RA; veinlets in pterostigmal area indistinct; two veins between pterostigma and RA; sc-r crossvein absent; RP originating posterior to ca. 1/8 wing length, with five main branches, branches at least bifurcate twice; two ra-rp crossveins; one gradate series (outer), with crossveins partly located distal to branching points; MA and MP bifurcate twice, with additional small forks near wing margin; two ma-mp crossveins; three m-cu crossveins; CuA with seven pectinate branches, mostly simple; CuP stem indistinct, distal possibly not fused with CuA or A1; three anal veins, all simple.

Abdomen apex covered with dense long setae, some setae longer than half abdomen width; ectoproct paired, posteriorly with long flat lobe-like projection, sternum 9 long, distally reaching half of the ectoproct lobe-like projection.

Genus *Trimantispa* Xiang, Chen and Yang gen. nov.

Type species: *Trimantispa poseidoni* Xiang, Chen and Yang gen. et sp. nov.

Etymology: The generic name is a combination of the prefix “*tri*-“ (Greek, meaning “three”) and “*mantispa*” (the type genus of the family Mantispidae), in reference to the trifurcate major process of the profemur. Gender feminine.

Diagnosis: Vertex with a pair of tubercles covered with long setae; pronotum tubularly elongated, robust (length/width ratio 2.69), equal in length to pterothorax, with paratergal lobes ventrally fused, lacking maculae. Forelegs raptorial, positioned at anterior part of pronotum; forecoxa longer than forefemur; forefemur shorter than combined length of foretibia and foretarsus, lacking posteroventral carina; processes saber-like, with conical Stitz organ at apex; major process located at basal 3/8 length of forefemur with branches at basal 1/5, trifurcate; foretibia straight, with ventral keel, ventrally with a row of reclined prostrate setae; foretarsus 5-segmented, tarsomeres 1–4 ventrally bearing with four, three, one, and two conical setae, basitarsus and tarsomere 5 longer, tarsomeres 2–4 subequal in length, basitarsus shorter than combined length of tarsomeres 2–4, pretarsus with a pair of simple claws, arolium triangular, and apically tapered. Mid- and hind legs long, combined length of femur and tibia greater than 0.6 of forewing length; basitarsus long, greater than 0.4 tibia length, more than 1.7 times longer than the combined length of tarsomeres 2–5. Wings narrow, length/width ratio greater than 3, with trichosors at apex, some trichosors in pterostigmal area multiple; humeral veinlet and subcostal veinlets simple; Sc fused with RA distally, pterostigma contacting RA, veinlets in pterostigmal area indistinct; pterostigma shape nearly trapezoidal; forewing with one sc-r crossvein, three ra-rp crossveins; two very short crossveins between M and R stems; CuP not contacting A1; three anal veins, distal ends simple; hind wing with Sc+RA fused distally; two ra-rp crossveins; one gradate series; one r-m crossvein between RP1 and MA; three m-cu crossveins; CuA with pectinate branches, branches distally bifurcate or simple; CuP stem absent; three anal veins, distal ends simple.

Remarks: *Trimantispa* gen. nov. exhibits a combination of features from the raptorial legs of *Doratomantispa* and *Acanthomantispa*, such as the major process located at basal 3/8 length of the forefemur with a median branch, similar to *Acanthomantispa*; the basal branching of the major process and the higher number of anteroventral and posteroventral processes with successively shorter lengths align with *Doratomantispa*. Overall, *Trimantispa* gen. nov. resembles *Doratomantispa* but also incorporates some features seen in *Acanthomantispa*, representing a hybrid of traits from both genera.

*Trimantispa poseidoni* Xiang, Chen and Yang gen. et sp. nov.

**Figure 7:**
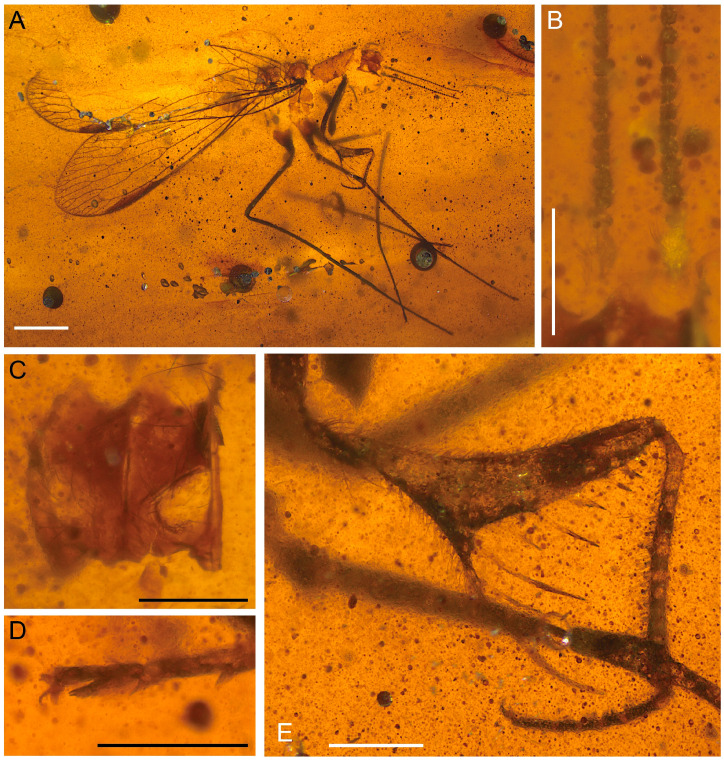
Holotype of *Trimantispa poseidoni* gen. et sp. nov., CNU-NEU-MA2018087. (**A**) Habitus, lateral view; (**B**) antennae; (**C**) vertex, dorsal view; (**D**) tarsus and pretarsus of hind leg; (**E**) foreleg. Scale bars = 2 mm (**A**), 0.5 mm (**B**–**E**).

**Figure 8:**
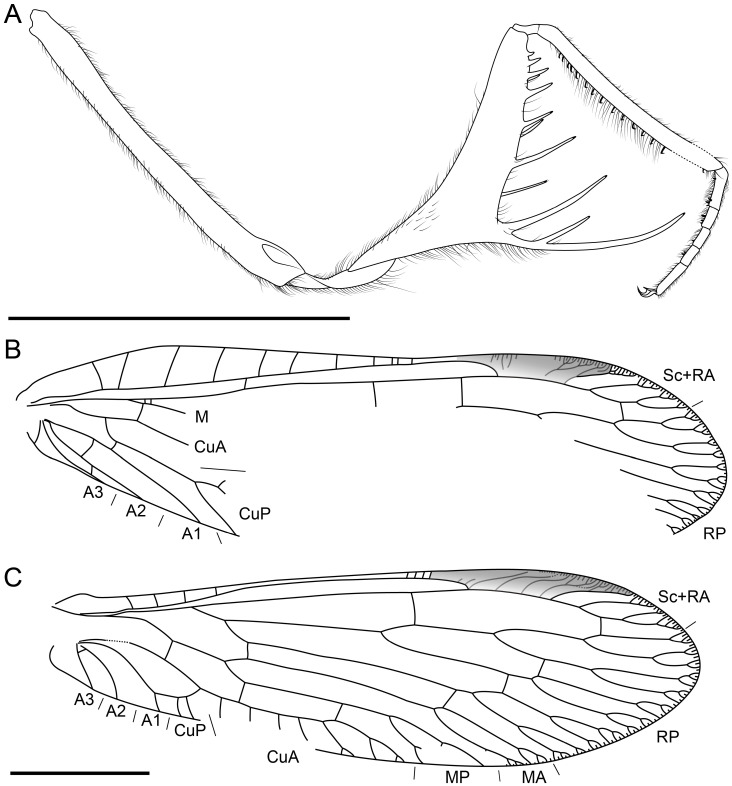
Holotype of *Trimantispa poseidoni* gen. et sp. nov., CNU-NEU-MA2018087. (**A**) Right foreleg, posterior view; (**B**) right forewing; (**C**) right hind wing. Scale bars = 2 mm.

Figure 7 and Figure 8.

Etymology: The specific epithet is named after Poseidon, the Greek god of the sea, whose weapon is a trident, in reference to the trifurcate major process of the forefemur.

Diagnosis: As for the genus.

Holotype: CNU-NEU-MA2018087, sex unknown, a relatively complete specimen with head and forewing damaged, abdomen not preserved.

Description: Vertex distinctly below compound eyes; compound eyes large, hemispherical, with straight ocular plane, parocular area concave; antenna length ca. 3/10 forewing length, scape distally swollen, length/width ratio ca. 2, pedicel length/width ratio 0.9, flagellum covered with sparse short setae, left antenna with 36 flagellomeres, right with 38, basal flagellomeres similar in size to pedicel, larger than distal flagellomeres; coronal suture distinct.

Pronotum tubularly elongated, covered with fine setae, lacking maculae; mesoscutum with round anterolateral margins. Forelegs raptorial, positioned at anterior part of pronotum; forecoxa elongated, covered with fine setae and pedicellate short setae; trochanter conical, dorsal surface with pedicellate thick setae; forefemur thickened, length/width ratio 4.58, basally elongated, dorsally and basally covered with long fine setae, closing surface with sparse setae, lacking posteroventral carina; forefemoral processes saber-like, with conical Stitz organ at the apex, anteroventral row with major process at basal 3/8 length of forefemur, trifurcate (two shorter branches, one at base, another at basal 1/5, with the basal one longer); three successively shorter processes between major process and forefemoral apex; posteroventral row with six successively shorter processes; foretibia straight, basally strongly curved, distally not produced, lacking tibial spur, with ventral keel, ventrally with a row of reclined prostrate setae (15 preserved) and long fine setae, dorsally with short setae; foretarsus 5-segmented, basitarsus and tarsomere 5 longer, ca. twice tarsomeres 2–4 individually, basitarsus slightly longer than tarsomere 5, tarsomeres 2–4 subequal in length, basitarsus shorter than combined length of tarsomeres 2–4; pretarsus with a pair of simple claws, arolium triangular, apically tapered.

Mid- and hind legs long, combined length of femur and tibia ca. 0.63 (mid leg) or 0.77 (hind leg) of forewing length; basitarsus extremely long, 0.45 (mid leg) or 0.41 (hind leg) of tibia length, 1.7–2 times much longer than combined length of tarsomeres 2–5; tarsomeres 2–5 successively shorter; tarsomeres 1–3 each with two setae, tarsomere 4 with four setae visible; pretarsus with a pair of simple claws, arolium rounded.

Wings elongate-oval (forewing length/width ratio 3.72, hind wing length/width ratio 3.2), with a single trichosor between the apex of two veinlets on the apical wing margin, and both single and multiple trichosors on the pterostigma margin, lacking nygmata and vesicae; preserved forewing without markings, humeral veinlet simple, costal space basally broad, apically tapered, subcostal veinlets simple, 11 present; one sc-r crossvein posterior to RP origin; pterostigma distinct, nearly trapezoidal, between C and Sc+RA, veinlets in pterostigmal area indistinct; three ra-rp crossveins, bifurcate distally with small forks or multifurcate near wing margin; one m-cu crossvein between M stem and CuA; Cu deeply forked, one cua-cup crossvein present; CuP with at least two branches; three anal veins, distal ends simple; cup-a1, a1–a2, and a2–a3 crossveins present. Hind wing without markings, humeral veinlet simple, costal space narrow, apically tapered, subcostal veinlets simple, ca. eight present; pterostigma distinct, nearly trapezoidal, between C and Sc+RA, Sc+RA distal branches distinguishable; sc-r crossvein absent; RP originating near ¼ wing length, with five main branches, branches bifurcate distally with small forks or multifurcate near wing margin; two ra-rp crossveins; one gradate series (outer), with gradate crossveins proximal to RP branch forking points; two r-m crossveins, one r-m between RP1 and MA; three m-cu crossveins; CuA with seven pectinate branches, branches with small forks near wing margin; CuP stem absent, distal not fused with A1, with two branches near wing margin; one cua-cup crossvein present; three anal veins, distal ends simple, with one a2–a3 crossvein.

Genus *Tribelomantispa* Xiang, Chen and Yang gen. nov.

Type species: *Tribelomantispa yangjiani* Xiang, Chen and Yang gen. et sp. nov.

Etymology: The generic name is a combination of the prefix “*tribel*-“ (Greek, meaning “three-pointed”) and “*mantispa*” (the type genus of the family Mantispidae), in reference to the trifurcate major process of the forefemur. Gender feminine.

Diagnosis: Pronotum tubularly elongated, robust (length/width ratio 2.42), equal in length to pterothorax, with paratergal lobes ventrally fused, lacking maculae. Forelegs raptorial, positioned at anterior part of pronotum; forecoxa longer than the forefemur, forefemur shorter than combined length of foretibia and foretarsus, lacking posteroventral carina; forefemoral processes saber-like, with conical Stitz organ at apex; major process located at basal 3/8 length of forefemur, trifurcate, with branches at basal 1/10 and 1/5; foretibia straight, with ventral keel, ventrally with a row of reclined prostrate setae; foretarsus 5-segmented, tarsomeres 1–4 ventrally bearing conical setae, basitarsus and tarsomere 5 longer, tarsomeres 2–4 subequal in length, basitarsus shorter than combined length of tarsomeres 2–4, pretarsus with a pair of simple claws. Mid- and hind legs long, combined length of femur and tibia greater than 0.5 of forewing length; basitarsus long, greater than 0.3 of tibia length, more than 1.5 times longer than combined length of tarsomeres 2–5. Wings narrow, length/width ratio greater than 3, with trichosors at apex, some trichosors in pterostigmal area multiple; subcostal veinlets simple; Sc not fused with RA distally, pterostigma not contacting RA, veinlets in pterostigmal area indistinct; pterostigma shape slender fusiform; forewing humeral veinlet simple, with one sc-r crossvein, three ra-rp crossveins; two gradate series, outer gradate incomplete; one very short crossvein between M and R stems; three m-cu crossveins; CuP not contacting A1; three anal veins, A1 with deep distal bifurcation, A2 and A3 simple; hind wing Sc not fused with RA distally; one gradate series; CuA with pectinate branches, branches distally bifurcate or simple.

Remarks: *Tribelomantispa* gen. nov. differs from *Tholomantispa* gen. nov. in the forecoxa longer than forefemur (equal to in *Tholomantispa* gen. nov.), the more distal position of the forefemoral major process (near basal 3/8 of forefemur in *Tribelomantispa* gen. nov., but proximal 3/16 in *Tholomantispa* gen. nov.), the presence of three branches on the major process (two in *Tholomantispa* gen. nov.), and the non-fusion of Sc with RA (fused in *Tholomantispa* gen. nov.). *Tribelomantispa* gen. nov. further differs from *Heteromantispa* gen. nov. by retaining a higher number of forefemoral processes in both anteroventral and posteroventral rows (processes strongly reduced in *Heteromantispa* gen. nov.) and by the presence of a distinct basal branch on the major process (median branch in *Heteromantispa* gen. nov.). *Tribelomantispa* gen. nov. is distinguished from *Trimantispa* gen. nov. by the following combination of characteristics: (1) Sc and RA not fused distally in both fore- and hind wings (fused in *Trimantispa* gen. nov.); (2) pterostigma slender and fusiform, not contacting RA (broader, nearly trapezoidal and contacting RA in *Trimantispa* gen. nov.); and (3) branches of the trifurcate forefemoral major process arising more distally, at ca. 1/10 and ca. 1/5 of the process length (at the very base and near ca. 1/5 in *Trimantispa* gen. nov.).

The raptorial leg morphology of *Tribelomantispa* gen. nov. is highly similar to that of *Trimantispa* gen. nov., with the major process located at the basal 3/8 length of the forefemur and branched at the base and middle in both genera. Like *Trimantispa* gen. nov., *Tribelomantispa* gen. nov. exhibits a blend of features from *Doratomantispa* and *Acanthomantispa*.

*Tribelomantispa yangjiani* Xiang, Chen and Yang gen. et sp. nov.

**Figure 9:**
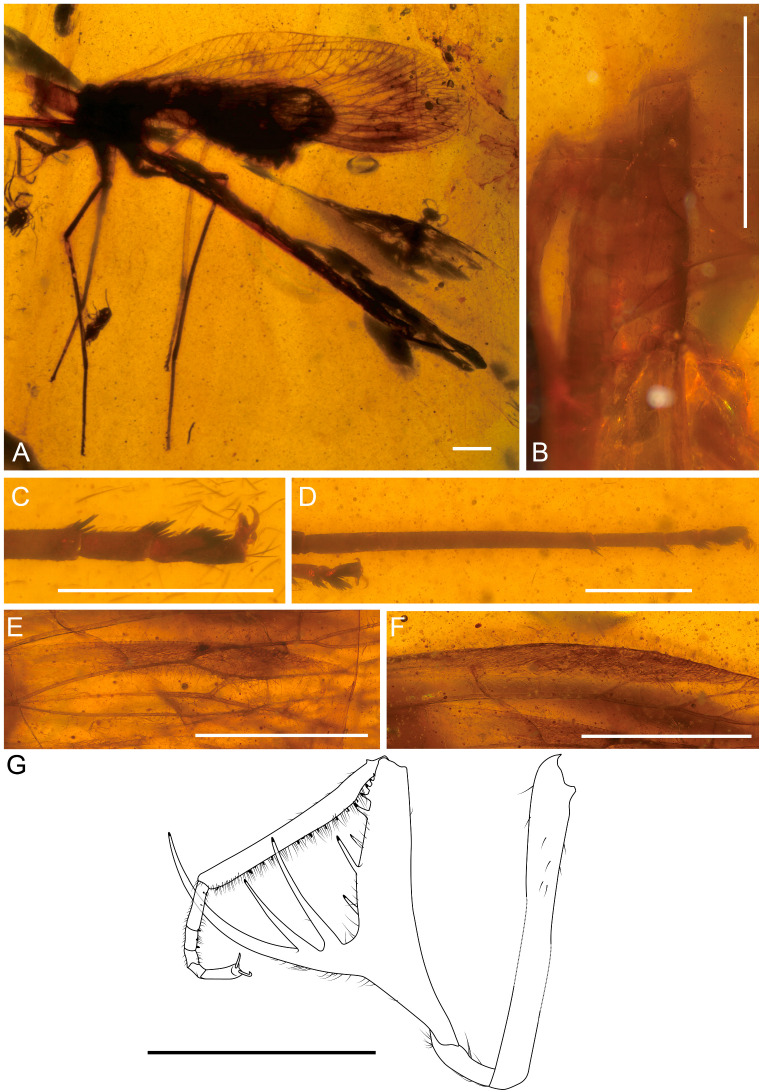
Holotype of *Tribelomantispa yangjiani* gen. et sp. nov., CNU-NEU-MA2018061. (**A**) Habitus, lateral view; (**B**) prothorax, lateral view; (**C**,**D**) left foreleg, posterior view; (**E**) left forewing pterostigma; (**F**) left hind wing pterostigma; (**G**) left foreleg, posterior view. Scale bars = 2 mm.

**Figure 10:**
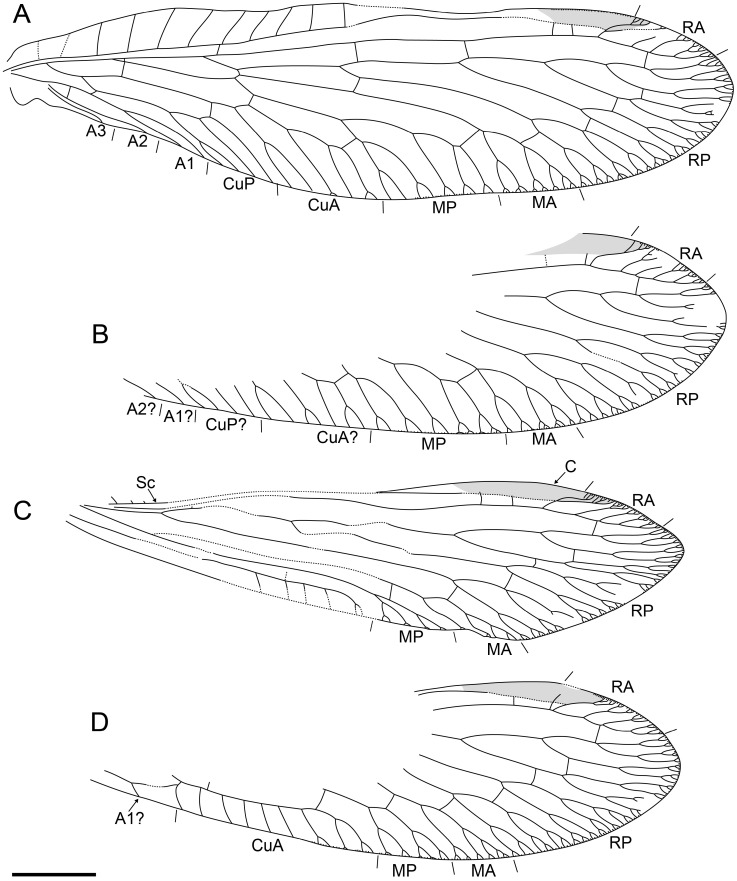
Holotype of *Tribelomantispa yangjiani* gen. et sp. nov., CNU-NEU-MA2018061. (**A**) Left forewing; (**B**) right forewing; (**C**) left hind wing; (**D**) right hind wing. Scale bar = 2 mm.

Figure 9 and Figure 10.

Etymology: The specific epithet is named after Yang Jian, a figure from ancient Chinese mythology, whose weapon is a three-pointed spear, in reference to the trifurcate major process of the forefemur.

Diagnosis: As for the genus.

Holotype: CNU-NEU-MA2018061, sex unknown, nearly complete specimen, head not preserved.

Description: Pronotum tubularly elongated, robust, with paratergal lobes ventrally fused, covered with short fine setae, lacking maculae; mesoscutum with round anterolateral margins. Forelegs raptorial, positioned at the anterior part of pronotum; forecoxa elongated, covered with sparse fine setae; trochanter conical, dorsal surface with sparse thick setae; forefemur thickened, length/width ratio 4.76, basally elongated and covered with long fine setae, closing surface with sparse setae, lacking posteroventral carina; forefemoral processes saber-like, with conical Stitz organ at apex, anteroventral row with major process at basal 3/8 length of forefemur, trifurcate, basal branch longer; three successively shorter processes between major process and forefemoral apex; posteroventral row with six successively shorter processes; foretibia straight, distally not produced, lacking tibial spur, with ventral keel, ventrally with a row of reclined prostrate setae (14 detected) and long fine setae, dorsally with sparse short setae; foretarsus 5-segmented, basitarsus and tarsomere 5 longer, ca. twice tarsomeres 2–4 individually, basitarsus slightly longer than tarsomere 5, tarsomeres 2–4 subequal in length, basitarsus shorter than combined length of tarsomeres 2–4 (ca. 0.8 times), tarsomeres 1–3 ventrally with four, two, and two conical setae, respectively; pretarsus with a pair of simple claws, arolium not visible.

Mid- and hind legs long, combined length of femur and tibia ca. 0.51 (mid leg) or 0.67 (hind leg) of forewing length; basitarsus extremely long, 0.41 (mid leg) or 0.33 (hind leg) of tibia length, 1.5–1.8 times longer than combined length of tarsomeres 2–5; tarsomeres 2–5 successively shorter; tarsomeres 1–3 with four, six, and eight setae, respectively, tarsomere 4 with 16 setae visible; pretarsus with a pair of simple claws, arolium rounded.

Wings elongate-oval (forewing length/width ratio 4.0, hind wing length/width ratio 3.4), with a single trichosor between the apex of two veinlets on the apical wing margin, and multiple trichosors on the basal wing margin, lacking nygmata and vesicae; forewing without markings, humeral veinlet simple, costal space basally broad, apically tapered, subcostal veinlets simple, nine visible; one sc-r crossvein posterior to RP origin; Sc terminating in pterostigmal area, distal end indistinct; pterostigma distinct, densely setose; veinlets in pterostigmal area indistinct, two veins between pterostigma and RA; RP originating posterior to 1/4 wing length, with six main branches, branches deeply bifurcate twice then multifurcate near wing margin; inner gradate series with only one crossvein, outer gradate crossveins distal to branch forking points; one very short crossvein between M and R stems; MA terminal forking similar to RP branches, with two main branches; MP subpectinately branched, with four main branches, branches distally bifurcate, with ma-mp crossvein; three m-cu crossveins, one m-cu between M stem and CuA; Cu deeply forked; one cua-cup crossvein between CuA distal stem and CuP distal branch; CuA subpectinately branched, with four main branches, branches distally bifurcate or simple; CuP with three pectinate branches; A2 and A3 simple; cup-a1 and a1-a2 crossveins present.

Hind wing without markings; pterostigma distinct, dense setae, nearly fusiform, not contacting RA; veinlets in pterostigmal area indistinct; two veins between pterostigma and RA, one in left hind wing extending to pterostigmal area; sc-r crossvein absent; RP with five main branches, branches deeply bifurcate twice then multifurcate near wing margin; one ra-rp crossvein visible; one gradate series (outer), with gradate crossveins distal to primary branch forking points of each RP branch; MA and MP with two main branches each; two m-cu crossveins visible; CuA with nine pectinate branches, most distal ends simple; A1 possibly simple. Abdomen not visible.

## 4. Discussion

The genus *Heteromantispa* gen. nov. possesses scale-like setae on its forewings, which are observed for the first time on mantispids. Among Neuroptera, scale-like setae are only known from Berothidae, Kalligrammatidae and Babinskaiidae to date [20,21,22]. In *Heteromantispa* gen. nov., scale-like setae are lanceolate, located on the main veins and subcostal veinlets at the forewing base. In Berothidae, scale-like setae are lanceolate with thin bases and apices, primarily along longitudinal veins, and in some taxa (e.g., *Podallea pellita* U. Aspöck & H. Aspöck, 1981 [23]) also on crossveins. In Kalligrammatidae, scale-like setae include elongate, spoon-shaped, flattened large forms along longitudinal veins and shorter, broad-based forms scattered across the wing surface [21]. In Babinskaiidae, scale-like setae are lanceolate on the proximal section of the forewing costal veins [22].

Scale-like setae are only present on females of Berothidae, which is the same as on *Xiaobabinskaia lepidotricha* in Babinskaiidae, although males of *X. lepidotricha* are not yet unknown. The holotype of *Heteromantispa* gen. et sp. nov. represents a male mantid lacewing bearing scale-like setae on the forewings, which is the first record of scale-like setae on the forewings of a male neuropteran. Scales are specialized setae and are present in many lineages of insects [24]. The scale-like setae, as well as scales, could act for functions including sensory perception, protection and thermoregulation [25]. For some phytophagous insects, the scale-like setae are also associated with gymnosperm seed plants [21]. In addition, female lacewings like Berothidae and Babinskaiidae may use the scale-like setae as a kind of organ related to chemical communication for attracting or searching mates [22]. However, this structure is found in the male *Heteromantispa*. This indicates that these scale-like setae may have other functions besides releasing pheromones.

Given that Mantispidae and Berothidae constitute Mantispoidea along with Rhachiberothidae and Dipteromantispidae [26], the presence of scale-like setae in both Mantispidae and Berothidae, as well as the absence in male Berothidae, implies a possibly complex evolutionary trajectory of this feature in the superfamily, but further analyses under a robust phylogenetic framework are needed.

Lu et al. established the subfamily Doratomantispinae based on phylogenetic reconstruction [11], where *Doratomantispa* and *Paradoxomantispa* form a distinct clade, supported by shared apomorphies such as the combined length of the forefemur and foretibia+foretarsus, an extremely long major process, successively shorter processes between the major process and the forefemoral apex, and a triangular arolium with a tapered apex. Additionally, Lu et al. described three extinct genera within the subfamily Drepanicinae: *Acanthomantispa*, *Dicranomantispa*, and *Psilomantispa* [11]. These three genera cluster together in the phylogenetic analysis of Lu et al. and do not form a monophyletic group with Doratomantispinae [11]. Beyond phylogenetic relationships, significant morphological differences exist between Doratomantispinae and the *Acanthomantispa* + *Dicranomantispa* + *Psilomantispa* clade. For instance, in Doratomantispinae, the major process of the forefemur is located at the base and branched only basally, with Sc and RA fused in both fore- and hind wings; in contrast, in *Acanthomantispa*, the major process is positioned near the mid-length of the forefemur with a median branch, the forefemur has a significant reduction in processes, and Sc and RA are not fused in the wings. However, in the recent study by Li et al. [26], *Doratomantispa*, *Paradoxomantispa*, *Pectispina*, *Acanthomantispa*, *Dicranomantispa,* and *Psilomantispa* form a clade that belongs to the Doratomantispinae subfamily. The findings of this study partially corroborate this result.

The newly described genera *Trimantispa* gen. nov. and *Tribelomantispa* gen. nov. exhibit transitional features between *Doratomantispa* (or *Tholomantispa* gen. nov.) and *Acanthomantispa* (or *Dicranomantispa*). This is elaborated as follows:

Firstly, *Trimantispa* gen. nov., *Tribelomantispa* gen. nov., *Doratomantispa*, and *Acanthomantispa* share numerous morphological features, many of which are unique to these genera and their related groups: (1) the major process of the forefemur is extremely long (far exceeding the forefemoral width); (2) the foretibia is straight with a row of reclined prostrate setae ventrally; (3) the foretarsus bears conical setae ventrally; (4) the pretarsus of the foreleg is simple with a triangular arolium that tapers apically; (5) the hind wing CuA is long and pectinate with at least seven branches; and (6) the hind wing CuP distal end is not fused with CuA or A1. Among these, except for features (5) and (6), the others were previously observed only in *Doratomantispa* or *Acanthomantispa* and their related groups.

Secondly, *Trimantispa* gen. nov. and *Tribelomantispa* gen. nov. exhibit a mix of characteristics from *Doratomantispa* and *Acanthomantispa*. Features consistent with *Trimantispa* gen. nov., *Tribelomantispa* gen. nov., and *Doratomantispa*, but differing from *Acanthomantispa*, include: (1) the major process has a basal branch (median branch in *Acanthomantispa*); (2) there are successively shorter ventral processes on the forefemur (with significant reduction in forefemoral processes in *Acanthomantispa*). Features consistent with *Trimantispa* gen. nov., *Tribelomantispa* gen. nov., and *Acanthomantispa*, but differing from *Doratomantispa*, include: (1) forecoxa longer than forefemur (equal to or shorter in *Doratomantispa*); (2) major process located near basal 3/8 length of forefemur with three branches (located at the base with two branches in *Doratomantispa*); (3) mid- and hind- leg basitarsus long, exceeding 1.5 times the combined length of tarsomeres 2–5 (basitarsus equal to or less than the combined length of tarsomeres 2–5 in *Doratomantispa*); (4) trichosors present only at the wing apex (*Doratomantispa* trichosors along the wing margin except at the base); and (5) subcostal veinlets simple and sparse (*Trimantispa* gen. nov. forewing 10.16 mm long with ca. 11 veinlets; *Tribelomantispa* gen. nov. forewing 17.17 mm long with ca. 15 veinlets; *Doratomantispa* subcostal veinlets relatively denser, e.g., *Doratomantispa zhuozhengmingi* Zhou et al. in Li et al., 2022, forewing 6.88 mm long with 13 veinlets). Additionally, the 1r-m crossvein in the hind wing of *Trimantispa* gen. nov. is located between RP1 and MA, consistent with *Acanthomantispa*, whereas in *Doratomantispa*, it is positioned between RP1 and the M stem.

Thirdly, differences between *Trimantispa* gen. nov. and *Tribelomantispa* gen. nov. mirror those between *Doratomantispa* and *Acanthomantispa*: (1) fusion of Sc and RA (fused in *Trimantispa* gen. nov. but unfused in *Tribelomantispa* gen. nov.); (2) pterostigma shape (nearly trapezoidal in *Trimantispa* gen. nov., slender fusiform in *Tribelomantispa* gen. nov.); and (3) position of forefemoral major process branch (basal and near basal 1/5 in *Trimantispa* gen. nov., basal 1/10 and 1/5 in *Tribelomantispa* gen. nov.). These differences are consistent and involve multiple characteristic systems, including wing venation, wing structure, and foreleg morphology, supporting their recognition as distinct genera rather than intraspecific variation, as they reflect fundamentally different morphological characteristics rather than continuous variation in a single characteristic. Among these, for features (2) and (3), *Trimantispa* gen. nov. resembles *Doratomantispa*, while *Tribelomantispa* resembles *Acanthomantispa*; for feature (1), both *Trimantispa* gen. nov. and *Doratomantispa* exhibit a basal branch, whereas the most basal branch in *Tribelomantispa* gen. nov. located at 1/10 is closer to the 1/6–1/5 branch position in *Acanthomantispa*.

## 5. Conclusions

This study significantly advances our understanding of Mantispidae by describing four new genera and four new species, along with one new combination from the Upper Cretaceous. Among these, *Tholomantispa* gen. nov. preserves the most complete male genitalia known for extinct mantispid species, offering critical insights into the structural details of gonocoxites and supporting the establishment of morphological characters for phylogenetic diagnosis. The discovery of scale-like setae on the forewings of *Heteromantispa polytricha* gen. et sp. nov. represents the first record of such structures in male Neuroptera, challenging previous assumptions about their distribution and suggesting potential secondary loss in extant taxa. Similarly, the documentation of processes on the forefemur across multiple genera introduces a novel morphological trait within Mantispidae, enriching our knowledge of their structural diversity.

The new genera *Trimantispa* gen. nov. and *Tribelomantispa* gen. nov. exhibit transitional morphologies between *Doratomantispa* and *Acanthomantispa*, supporting their placement within one clade while highlighting a complex combination of shared and derived characters.

## Data Availability

The original contributions presented in this study are included in the article. Further inquiries can be directed to the corresponding author.

## Abbreviations

The following abbreviations are used in this manuscript:

A1–A3	First to third branches of anal vein
C	Costa
Cu	Cubitus
CuA	Cubitus anterior
CuP	Cubitus posterior
MA	Media anterior
MP	Media posterior
R	Radius
RA	Radius anterior
RP	Radius posterior
RP1	Proximal-most branch of RP
Sc	Subcosta
